# Supplementary material for: A Critical View of the Application of the APEX Software (Aqueous Photochemistry of Environmentally-Occurring Xenobiotics) to Predict Photoreaction Kinetics in Surface Freshwaters
Source: Molecules. 2019 Dec 18;25(1):9. doi: 10.3390/molecules25010009 (PMC7017383; doi:10.3390/molecules25010009)
Supplement: Supplementary file 1 [file molecules-25-00009-s001.zip › 3.2.4_gcc-4.4.0.zip!/3.2.4_gcc-4.4.0/3.2.4_gcc-4.4.0/doc/octave/html/interpreter/Graphics-Objects.html]

Graphics Objects - Untitled


Next: Graphics Object Properties,
Up: Advanced Plotting


---

#### 15.2.1 Graphics Objects

Plots in Octave are constructed from the following graphics
objects. Each graphics object has a set of properties that define its
appearance and may also contain links to other graphics objects.
Graphics objects are only referenced by a numeric index, or handle.

root figure: The parent of all figure objects. The index for the root figure is defined to be 0. figure: A figure window. axes: A set of axes. This object is a child of a figure object and may be a parent of line, text, image, patch, or surface objects. line: A line in two or three dimensions. text: Text annotations. image: A bitmap image. patch: A filled polygon, currently limited to two dimensions. surface: A three-dimensional surface.

To determine whether a variable is a graphics object index or a figure
index, use the functions `ishandle` and `isfigure`.

— Built-in Function: **ishandle** (h)  
> Return true if h is a graphics handle and false otherwise.

— Function File: **ishghandle** (h)  
> Return true if h is a graphics handle and false otherwise.

— Function File: **isfigure** (h)  
> Return true if h is a graphics handle that contains a figure
> object and false otherwise.

The function `gcf` returns an index to the current figure object,
or creates one if none exists. Similarly, `gca` returns the
current axes object, or creates one (and its parent figure object) if
none exists.

— Function File: **gcf** ()  
> Return the current figure handle. If a figure does not exist, create
> one and return its handle. The handle may then be used to examine or
> set properties of the figure. For example,
>
> ```
>           fplot (@sin, [-10, 10]);
>           fig = gcf ();
>           set (fig, "visible", "off");
> ```
>
> plots a sine wave, finds the handle of the current figure, and then
> makes that figure invisible. Setting the visible property of the
> figure to `"on"` will cause it to be displayed again.
>
>
>
> **See also:** get, set.

— Function File: **gca** ()  
> Return a handle to the current axis object. If no axis object
> exists, create one and return its handle. The handle may then be
> used to examine or set properties of the axes. For example,
>
> ```
>           ax = gca ();
>           set (ax, "position", [0.5, 0.5, 0.5, 0.5]);
> ```
>
> creates an empty axes object, then changes its location and size in
> the figure window.
>
>
>
> **See also:** get, set.

The `get` and `set` functions may be used to examine and set
properties for graphics objects. For example,

```
     get (0)
         => ans =
            {
              type = root
              currentfigure = [](0x0)
              children = [](0x0)
              visible = on
     			...
            }
```

returns a structure containing all the properties of the root figure.
As with all functions in Octave, the structure is returned by value, so
modifying it will not modify the internal root figure plot object. To
do that, you must use the `set` function. Also, note that in this
case, the `currentfigure` property is empty, which indicates that
there is no current figure window.

The `get` function may also be used to find the value of a single
property. For example,

```
     get (gca (), "xlim")
         => [ 0 1 ]
```

returns the range of the x-axis for the current axes object in the
current figure.

To set graphics object properties, use the set function. For example,

```
     set (gca (), "xlim", [-10, 10]);
```

sets the range of the x-axis for the current axes object in the current
figure to [-10, 10]. Additionally, calling set with a graphics
object index as the only argument returns a structure containing the
default values for all the properties for the given object type. For
example,

```
     set (gca ())
```

returns a structure containing the default property values for axes
objects.

— Built-in Function: **get** (h, p)  
> Return the named property p from the graphics handle h.
> If p is omitted, return the complete property list for h.
> If h is a vector, return a cell array including the property
> values or lists respectively.

— Built-in Function: **set** (h, p, v, ...)  
> Set the named property value or vector p to the value v
> for the graphics handle h.

— Function File: parent = **ancestor** (h, type)  
— Function File: parent = **ancestor** (h, type, 'toplevel')  
> Return the first ancestor of handle object h whose type matches
> type, where type is a character string. If type is a
> cell array of strings, return the first parent whose type matches
> any of the given type strings.
>
> If the handle object h is of type type, return h.
>
> If `"toplevel"` is given as a 3rd argument, return the highest
> parent in the object hierarchy that matches the condition, instead
> of the first (nearest) one.
>
>
>
> **See also:** get, set.

— Function File: h = **allchild** (handles)  
> Find all children, including hidden children, of a graphics object.
>
> This function is similar to `get (h, "children")`, but also
> returns includes hidden objects. If handles is a scalar,
> h will be a vector. Otherwise, h will be a cell matrix
> of the same size as handles and each cell will contain a
> vector of handles.
>
>
>
> **See also:** get, set, findall, findobj.

You can create axes, line, and patch objects directly using the
`axes`, `line`, and `patch` functions. These objects
become children of the current axes object.

— Function File: **axes** ()  
— Function File: **axes** (property, value, ...)  
— Function File: **axes** (h)  
> Create an axes object and return a handle to it.

— Function File: **line** ()  
— Function File: **line** (x, y)  
— Function File: **line** (x, y, z)  
— Function File: **line** (x, y, z, property, value, ...)  
> Create line object from x and y and insert in current
> axes object. Return a handle (or vector of handles) to the line
> objects created.
>
> Multiple property-value pairs may be specified for the line, but they
> must appear in pairs.

— Function File: **patch** ()  
— Function File: **patch** (x, y, c)  
— Function File: **patch** (x, y, z, c)  
— Function File: **patch** (fv)  
— Function File: **patch** ('Faces', f, 'Vertices', v, ...)  
— Function File: **patch** (..., prop, val)  
— Function File: **patch** (h, ...)  
— Function File: h = **patch** (...)  
> Create patch object from x and y with color c and
> insert in the current axes object. Return handle to patch object.
>
> For a uniform colored patch, c can be given as an RGB vector,
> scalar value referring to the current colormap, or string value (for
> example, "r" or "red").
>
> If passed a structure fv contain the fields "vertices", "faces"
> and optionally "facevertexcdata", create the patch based on these
> properties.

— Function File: **fill** (x, y, c)  
— Function File: **fill** (x1, y1, c1, x2, y2, c2)  
— Function File: **fill** (..., prop, val)  
— Function File: **fill** (h, ...)  
— Function File: h = **fill** (...)  
> Create one or more filled patch objects, returning a patch object for each.

— Function File: **surface** (x, y, z, c)  
— Function File: **surface** (x, y, z)  
— Function File: **surface** (z, c)  
— Function File: **surface** (z)  
— Function File: **surface** (..., prop, val)  
— Function File: **surface** (h, ...)  
— Function File: h = **surface** (...)  
> Plot a surface graphic object given matrices x, and y from
> `meshgrid` and a matrix z corresponding to the x and
> y coordinates of the surface. If x and y are vectors,
> then a typical vertex is (x(j), y(i), z(i,j)). Thus,
> columns of z correspond to different x values and rows of
> z correspond to different y values. If x and y
> are missing, they are constructed from size of the matrix z.
>
> Any additional properties passed are assigned to the surface.
>
>
>
> **See also:** surf, mesh, patch, line.

By default, Octave refreshes the plot window when a prompt is printed,
or when waiting for input. To force an update at other times, call the
`drawnow` function.

— Built-in Function: **drawnow** ()  
— Built-in Function: **drawnow** ("expose")  
— Built-in Function: **drawnow** (term, file, mono, debug\_file)  
> Update figure windows and their children. The event queue is flushed and
> any callbacks generated are executed. With the optional argument
> `"expose"`, only graphic objects are updated and no other events or
> callbacks are processed.
> The third calling form of `drawnow` is for debugging and is
> undocumented.

Only figures that are modified will be updated. The `refresh`
function can also be used to force an update of the current figure, even if
it is not modified.

— Function File: **refresh** ()  
— Function File: **refresh** (h)  
> Refresh a figure, forcing it to be redrawn. Called without an
> argument the current figure is redrawn, otherwise the figure pointed
> to by h is redrawn.
>
>
>
> **See also:** drawnow.

Normally, high-level plot functions like `plot` or `mesh` call
`newplot` to initialize the state of the current axes so that the
next plot is drawn in a blank window with default property settings. To
have two plots superimposed over one another, use the `hold`
function. For example,

```
     hold on;
     x = -10:0.1:10;
     plot (x, sin (x));
     plot (x, cos (x));
     hold off;
```

displays sine and cosine waves on the same axes. If the hold state is
off, consecutive plotting commands like this will only display the last
plot.

— Function File: **newplot** ()  
> Prepare graphics engine to produce a new plot. This function should
> be called at the beginning of all high-level plotting functions.

— Function File: **hold**  
— Function File: **hold** state  
— Function File: **hold** (hax, ...)  
> Toggle or set the 'hold' state of the plotting engine which determines
> whether new graphic objects are added to the plot or replace the existing
> objects.
>
> `hold on`: Retain plot data and settings so that subsequent plot commands are displayed on a single graph. `hold off`: Clear plot and restore default graphics settings before each new plot command. (default). `hold`: Toggle the current 'hold' state.
>
> When given the additional argument hax, the hold state is modified
> only for the given axis handle.
>
> To query the current 'hold' state use the `ishold` function.
>
>
>
> **See also:** ishold, cla, newplot, clf.

— Function File: **ishold**  
> Return true if the next line will be added to the current plot, or
> false if the plot device will be cleared before drawing the next line.

To clear the current figure, call the `clf` function. To clear the
current axis, call the `cla` function. To bring the current figure
to the top of the window stack, call the `shg` function. To delete
a graphics object, call `delete` on its index. To close the
figure window, call the `close` function.

— Function File: **clf** ()  
— Function File: **clf** ("reset")  
— Function File: **clf** (hfig)  
— Function File: **clf** (hfig, "reset")  
> Clear the current figure window. `clf` operates by deleting child
> graphics objects with visible handles (`HandleVisibility` = on).
> If hfig is specified operate on it instead of the current figure.
> If the optional argument `"reset"` is specified, all objects including
> those with hidden handles are deleted.
>
>
>
> **See also:** cla, close, delete.

— Function File: **cla** ()  
— Function File: **cla** ("reset")  
— Function File: **cla** (hax)  
— Function File: **cla** (hax, "reset")  
> Delete the children of the current axes with visible handles.
> If hax is specified and is an axes object handle, operate on it
> instead of the current axes. If the optional argument `"reset"`
> is specified, also delete the children with hidden handles.
>
>
>
> **See also:** clf.

— Function File: **shg**  
> Show the graph window. Currently, this is the same as executing
> `drawnow`.
>
>
>
> **See also:** drawnow, figure.

— Function File: **delete** (file)  
— Function File: **delete** (handle)  
> Delete the named file or graphics handle.
>
> Deleting graphics objects is the proper way to remove
> features from a plot without clearing the entire figure.
>
>
>
> **See also:** clf, cla.

— Command: **close**  
— Command: **close** (n)  
— Command: **close** all  
— Command: **close** all hidden  
> Close figure window(s) by calling the function specified by the
> `"closerequestfcn"` property for each figure. By default, the
> function `closereq` is used.
>
>
>
> **See also:** closereq.

— Function File: **closereq** ()  
> Close the current figure and delete all graphics objects associated
> with it.
>
>
>
> **See also:** close, delete.
